# Buruli Ulcer in Traveler from Suriname, South America, to the Netherlands

**DOI:** 10.3201/eid2103.141237

**Published:** 2015-03

**Authors:** William R. Faber, Bouke de Jong, Henry J.C. de Vries, Jim E. Zeegelaar, Françoise Portaels

**Affiliations:** Academic Medical Center, Amsterdam, the Netherlands (W.R. Faber, H.J.C. de Vries);; Public Health Service of Amsterdam, Amsterdam (W.R. Faber, H.J.C. de Vries);; Institute of Tropical Medicine, Antwerp, Belgium (B. de Jong, F. Portaels);; Flevohospital, Almere, the Netherlands (J.E. Zeegelaar)

**Keywords:** Mycobacterium ulcerans, Buruli ulcer, bacteria, antibiotics, antimicrobial drugs, Suriname, South America, latent infection, the Netherlands, tuberculosis and other mycobacteria

## Abstract

We report Buruli ulcer in a man in the Netherlands. Phenotyping of samples indicate the Buruli pathogen was acquired in Suriname and activated by trauma on return to the Netherlands. Awareness of this disease by clinicians in non–Buruli ulcer–endemic areas is critical for identification.

Buruli ulcer (BU) is a potentially disabling affliction of inhabitants of tropical wetlands (*1*) caused by *Mycobacterium ulcerans* and characterized by necrosis of skin, subcutaneous tissue, and sometimes bone. Major foci are in West and Central Africa and minor endemic foci in Australia, Southeast Asia, Mexico, and South America.

BU is rare in South America; 15 cases were reported in Peru since 1996 (*1*; H. Guerra, pers. comm.), and 242 cases were reported during 1969–2011 in French Guiana (*2*), which is bordered by Suriname to the west. Mycobacterial ulcers have been seen in Suriname in the past (R.F.M. Lai, A. Fat, L. Sabajo, pers. comm.), but these cases were not confirmed by laboratory tests. We report a case of BU in a patient from Suriname diagnosed in the Netherlands.

## The Case-Patient

In February 2013, a 70-year-old man sought care at the University of Amsterdam Department of Dermatology for a neuropathic ulcer on his left foot. It was determined that he had a neuropathic ulcer, a complication of nerve damage caused by leprosy, for which he was treated successfully. Data from his medical history were recovered and reexamined at this time.

He was a native of Suriname, where he lived until he moved to the Netherlands 1977. He had multiple instances of treatment for skin infections, beginning with a diagnosis of leprosy (Hansen disease, caused by the mycobacterium *M. leprae*) when he was 10 years of age. 

He was treated continuously with dapsone during 1952–1978. In 1977, he moved to Groningen, the Netherlands, and clofazimine was added to his treatment for leprosy for 6 months. In February 1982, he moved to Amsterdam and sought routine follow-up care for leprosy at the University of Amsterdam Department of Dermatology, and quiescent borderline lepromatous leprosy was diagnosed. He was treated during February 1982–February 1983 with a triple therapy consisting of rifampin, dapsone, and clofazimine.

In 1984, the man traveled to Paramaribo and Coronie in Suriname, where he stayed for 4 weeks during the spring season. During that time, he fished in creeks. In December 1984, he sought treatment again at the University of Amsterdam Department of Dermatology for a painful red swelling on the dorsal side of the right wrist after he had scratched it on a rough wall 5 weeks earlier, in mid-November. Beginning in February 1985, the patient had signs of infection of the wound, including conspicuous edematous swelling of the hand. Radiographic imaging showed no signs of osteomyelitis. The patient was treated with flucloxacillin; the symptoms subsided. However, during April 26–May 11, 1985, the patient experienced recurrent infection with abscess formation. A biopsy was taken, and an acid-fast bacilli (AFB) smear and culture for nontuberculous mycobacteria were done. The histopathology report indicated scarring dermis and mixed cell infiltrate, leucocytoclastic vasculitis, fibrinoid changes, and some diffusely spread histiocytes. AFB staining was negative. On June 3, partly dry wounds, 1 deep ulcer, fistula formation, and spontaneous drainage of another lesion were documented. The wound was cultured, and amoxicillin and clavulinic acid 4 times daily for 3 weeks was prescribed; after that time, the patient’s treatment for related conditions consisted of local wound care. After 6 weeks’ incubation, the mycobacterial culture was positive at 30°C but negative at 37°C and 45°C. In August, ulcers were still present, and multiple nodules at the underarm and an enlarged lymph node at the elbow were palpable. Infection with *M. ulcerans* was suspected clinically.

On January 20, 1986, the isolate was sent to the Institute of Tropical Medicine of Antwerp and was identified as *M. ulcerans* ITM 842 according to phenotypic characteristics (*3*). The isolate was further analyzed by several genotypic methods (*3*–*6*). By April 7, 1986, the ulcers were nearly completely closed. 

## Conclusions

We describe a patient infected with *M. ulcerans* strain ITM 842. Phenotypic characteristics of isolates from Suriname and French Guiana are identical, whereas isolates originating in Africa, Australia, and Asia have different phenotypic characteristics (*3*). Variable-number tandem-repeat genotypic analysis had been shown to discriminate the Suriname isolate from French Guiana isolates and from mycolactone-producing *M. marinum* (*4*,*5*). Results confirmed that this isolate was different from *M. ulcerans* isolates from other geographic origins, albeit closely related to *M. ulcerans* strains from French Guiana by genetic characteristics (*4*,*5*). These results suggest that the patient acquired his infection in Suriname.

The incubation period of primary BU is estimated to be 2–3 months, but latency of the disease can span years. Persons who were in an area to which BU is endemic months to years earlier can manifest BU in a traumatized body site (*6**,**7*).

Co-infections with leprosy and BU have been described, but rarely (*8*). During 1952–1977, the patient was treated with dapsone monotherapy. Additionally, he received clofazimine for 6 months upon his arrival in the Netherlands. Subsequently, during February 1982–February 1983, he was treated with a triple therapy consisting of rifampin, dapsone, and clofazimine. Treatment of BU patients with dapsone has not been properly evaluated in humans (*9*), and clofazimine is not indicated in the treatment of patients with BU (*10*); however, rifampin is highly bactericidal against this organism (*11*).

If the patient was infected with *M. ulcerans* before his arrival in the Netherlands in 1977, it is unlikely that the dapsone and clofazimine that he received for treatment of leprosy could have controlled the infection. Whether or not he had been infected with *M. ulcerans* when he first arrived in the Netherlands, treatment for 1 year with rifampin and dapsone before he returned to Suriname for 4 weeks in 1984 would have been expected to have killed any *M. ulcerans*. Thus, the most likely hypothesis is that he acquired BU infection during the 4 weeks he spent in Suriname in 1984, and that the scratch obtained against a rough wall in November 1984 in the Netherlands reactivated a latent infection. It is unlikely that the rough wall was contaminated by *M. ulcerans*, because BU is not considered prevalent in Europe. 

There are many similarities between this case and that reported by Lindo and Daniels (*7*). Both patients manifested symptoms of BU after having left a disease-endemic area, and in both patients, the lesion developed at the site of a trauma that occurred in a non–disease-endemic country.

Correct laboratory confirmation remains essential to confirm the discovery of new foci of BU, and the use of genetic mapping can provide additional information about the geographical location in which patients were infected. The importance of laboratory confirmation of BU has been emphasized by the World Health Organization’s manual for the laboratory diagnosis of BU (*12*).The study of imported cases, and in particular this case from Suriname, has enabled us to acquire more knowledge regarding latency and reactivation of the disease, and the existence of new foci. As in the report by Meyers et al (*13*), this case report emphasizes the role of trauma to the skin in the delayed manifestation of this disease caused by *M. ulcerans*.

Reports from European countries, Canada, America, and Australia describe travelers visiting friends or relatives in BU-endemic countries who manifested BU upon return to a non–disease-endemic area (*14*,*15*). Thus, BU may be diagnosed in patients late, or not diagnosed, in non–disease-endemic countries, where health care professionals are usually not familiar with the condition or its causal organism. Delayed diagnosis often leads to severe disabilities. In countries where BU is uncommon, the clinician’s consideration of the patient’s recent travel history and awareness of cutaneous and bone lesions of BU are needed for accurate testing, diagnosis, and treatment of the patient.
